# Stronger feelings of loneliness among Moroccan and Turkish older adults in the Netherlands: in search for an explanation

**DOI:** 10.1007/s10433-020-00562-x

**Published:** 2020-02-22

**Authors:** Theo G. van Tilburg, Tineke Fokkema

**Affiliations:** 1 Department of Sociology, Vrije Universiteit Amsterdam, Amsterdam, The Netherlands; 2 Netherlands Interdisciplinary Demographic Institute, The Hague, The Netherlands; 3 University of Groningen, Groningen, The Netherlands; 4 Department of Public Administration and Sociology, Erasmus University Rotterdam, Rotterdam, The Netherlands

**Keywords:** Loneliness, Older migrants, Measurement equivalence, Risk factors

## Abstract

The prevalence of loneliness among Turkish–Dutch and Moroccan–Dutch older adults is higher than among Dutch older adults of non-migrant origin. Three explanations may account for this difference: (1) differential item functioning might result in scores that vary in intensity and in meaning across categories; (2) the position of migrants is much more vulnerable than that of non-migrants; (3) the lack of protective factors has more severe consequences for older migrants. The Longitudinal Aging Study Amsterdam interviewed 176 persons born in Morocco and 235 born in Turkey, aged 55–66 and living in urban areas. They are compared with a matched sample of 292 Dutch persons. The psychometric properties of the loneliness scale are satisfying, although there is some differential item functioning. Older migrants have more frequent social contacts but are at a disadvantage in other domains. Taking into account differences in social participation, satisfaction with income, mastery and depressive symptoms, the difference between older migrants’ and non-migrants’ loneliness is reduced by more than half. Protective factors are equally important for older migrants and non-migrants. Exceptions are marriage (less protective for Moroccans), frequent contact with children/children-in-law (mostly for Turks), a higher educational level (protects Moroccans and Turks) and better physical functioning (less for Turks). Being an older migrant and belonging to a minority might further contribute to feelings of loneliness. Interventions can be directed at stimulating social contact, but also at aspects like enhancing the appreciation of their social status and avoiding negative interpretations of a situation.

**Electronic supplementary material:**

The online version of this article (10.1007/s10433-020-00562-x) contains supplementary material, which is available to authorized users.

## Introduction

Compared to people without a migration background, loneliness among migrants is common (Ajrouch 2008; Fokkema and Naderi 2013; Vancluysen and Van Craen 2011). Prevalence among migrants varies depending on their origin. In England, the prevalence is high among older adults from Pakistan, Bangladesh, China, Africa and the Caribbean, but not among those of Indian origin (Victor et al. 2012). In Canada, loneliness among older migrants from a different linguistic and cultural background is above average, in contrast to migrants who have many similarities with Canadian-born older adults (de Jong Gierveld et al. 2015). Older migrants in Canada are more lonely, but not when they identify themselves as British or French (Wu and Penning 2015).

Twenty years ago, there were already signs of strong loneliness among older migrants in the Netherlands, but the image of isolation and loneliness was considered as a possible cliché, fed by harrowing individual cases (Tesser et al. 1998). However, data since 2010 show relatively high levels of loneliness among older migrants in the four big Dutch cities (Amsterdam, Rotterdam, The Hague and Utrecht) (el Fakiri and Bouwman-Notenboom 2015; Uysal-Bozkir et al. 2017). Older migrants of Turkish origin are particularly lonely, followed by those of Moroccan and Surinamese origin. The factors related to these differences have not yet been investigated in detail.

In this study, we examine whether differences in loneliness between people aged 55–66 of Moroccan, Turkish and Dutch origin can be understood based on three factors: in comparison with non-migrants, migrants (1) have a different understanding of the concept of loneliness and report loneliness relatively quickly; (2) have higher loneliness-related risks; and (3) suffer more severe consequences when protective factors are lacking. For the sake of conciseness and readability, we mostly refer to the categories of origin as Moroccan, Turkish and Dutch.

### The concept and measurement of loneliness

Loneliness is a negative feeling in a situation of loss and dissatisfaction with the social network. It is the outcome of a process in which a person weighs up his existing personal relationships against his own wishes and social expectations with regard to relationships. If the network of relationships is too small, or the relationships are of insufficient quality, there is often loneliness (de Jong Gierveld et al. 2018). People have a strong need for social relationships in which they find connection, affection and involvement (Baumeister and Leary 1995). Loneliness is a social and historical way of understanding relationships (Bound Alberti 2018), but the core of loneliness is seen as a universal human experience because the need for social relationships is fundamental (Perlman 2004).

Several psychometric studies show that loneliness is measured equivalently in Western and non-Western countries (Durak and Senol-Durak 2010; Hawkley et al. 2012), in several Western countries (van Tilburg et al. 2004) and European countries (de Jong Gierveld and van Tilburg 2010), and among migrants and non-migrants in the Netherlands (Uysal-Bozkir et al. 2017; Visser and el Fakiri 2016). Qualitative research among Dutch residents of Turkish and Surinamese origin (Torensma 2014) indicates sufficient construct validity, although the concept of emptiness points to a lack of feeling of connection with God among older Turks and a lack of social relationships among older Surinamese. It also seems that older Turks have a lower threshold for agreeing to a loneliness item about friendship (Leung and Bond 1989; Victor et al. 2012). Given the repeatedly found cross-national and cross-cultural equivalence of loneliness, Hypothesis 1 is that loneliness of older migrants and non-migrants can be compared using an existing measurement instrument.

### High risks among migrants

Many factors increase the risk of loneliness. For example, older adults without a partner, have few persons they maintain frequent contact with, lack paid or voluntary work, do not participate in social activities, or have a low income or poor health are at an increased risk of loneliness (Fokkema et al. 2012; Hansen et al. 2009). Older Moroccan and Turkish migrants have many more risk factors than non-migrants, but not in all domains (Schellingerhout 2004; Steinbach 2018). That older migrants co-reside with children more often protects. However, because their contacts outside the home are often with relatives, their network is homogeneous. They are often low-skilled and usually have a low income. Many started working at a very young age. Later, in the Netherlands, men mostly did physically heavy work under poor working conditions and were either long-term unemployed or prematurely incapacitated for work. It is common for women to never have worked outside the home, but they tend to be the anchor of usually large families. Partly because of language barriers and cultural differences, older migrants can find it difficult to connect with Dutch society (including regular welfare and care institutions) and to take control of their own lives—i.e. they have low mastery. They report a high proportion of chronic conditions and physical limitations in terms of mobility and personal care, as well as a low level of perceived health. Hypothesis 2 is that the stronger loneliness among older migrants compared to non-migrants is partly attributable to the higher risks among older migrants.

### Differential risk and protective effects

Risk and protective factors may carry a different weight for older migrants than for non-migrants, hence affecting the impact on loneliness. Perhaps the most obvious example is the higher importance that older Moroccan and Turkish migrants attach to the family, in particular their children. This protective factor interacts with the risk factor that they, as members of a minority ethnic group, are more likely to experience discrimination (Pettigrew et al. 1997) and social exclusion (de Tavernier and Draulans 2019). This firstly leads to what is known as minority stress (du Plooy et al. 2019; Meyer 2003); social security within the family may help cope with this stress. Secondly, it results in few inter-ethnic meeting opportunities; a lack of alternative social ties increases the need for close and supportive bonds within the family. Their dependency on the family is further reinforced by the resistance of older Moroccans and Turks to professional care and to their adherence to the filial responsibility expectations that are part of their collectivistic culture (Conkova and Lindenberg 2018). One might thus assume that being strongly embedded in the family serves as a buffer against loneliness, especially for older Moroccans and Turks. Conversely, those who do not have good contact with family members are likely to be at a greater risk of loneliness than non-migrants. It remains to be seen whether the impact of lacking other resources (i.e. education, income, mastery, health) on loneliness is stronger for older Moroccans and Turks than for their native peers. Hypothesis 3 is that older migrants are on average lonelier than non-migrants partly because the risk factors and the lack of protective factors weigh more heavily on them.

## Method

### Respondents

The data were from the Longitudinal Aging Study Amsterdam (Huisman et al. 2011). The samples were stratified by gender and taken from municipal population registers. In 2012 and 2013, 1023 men and women born between 1948 and 1957 were interviewed—all residents of Amsterdam, Zwolle, Oss, or six surrounding municipalities. The response rate was 63. Almost all were of Dutch origin. In 2013 and 2014, 209 persons of Moroccan origin and 269 of Turkish origin were interviewed. They were residents of Alkmaar, Almere, Amersfoort, Amsterdam, Breda, Eindhoven, Enschede, Haarlem, Helmond, Hilversum, Nijmegen, Oss, Tilburg, Zaanstad and Zwolle. The response rate was 45.

The interviews were conducted in Dutch, Moroccan-Arabic (Darija), Berber (Tarifit) or Turkish. For various questions, the translations were taken from previous research, such as the loneliness scale (Uysal-Bozkir et al. 2017) and the CES-D depression scale (Spijker et al. 2004). Questions unavailable in Moroccan-Arabic, Berber or Turkish were translated by two professionals using the back translation method.

To harmonise the samples, older adults living independently in urban neighbourhoods; married and cohabiting with their partner or unmarried and without a partner; and born in the Netherlands (*N* = 292), Morocco (*N* = 176) or Turkey (*N* = 235) were selected. On average, the migrants had been in the Netherlands since 1977.

### Measuring instruments

Loneliness was measured using the 11-item scale of De Jong Gierveld (de Jong Gierveld and van Tilburg 1999). An answer ‘yes’ to six negatively formulated items, an answer ‘no’ to five positively formulated items, and an answer in the category ‘more or less’ count as a loneliness point. Scale values are 0–11. Two direct measurements of loneliness were included to indicate concurrent validity: the statement ‘I sometimes feel lonely’ with the same answer possibilities as the scale items, and whether the respondents count themselves among the not (1), moderate (2), strong (3) or very strong (4) lonely ones. Table 1 shows the descriptive data.

**Table 1:** Descriptive data (mean or proportion) by origin

	Dutch		Moroccan		Turkish		Dutch versus Moroccan	Dutch versus Turkish	Moroccan versus Turkish
	*N* = 292		*N* = 176		*N* = 235				
	M	SD	M	SD	M	SD			
Loneliness (scale score, 0–11)	1.7	2.6	4.5	3.1	5.6	3.3	***	***	***
I sometimes feel lonely (1–3)	1.5	0.7	1.7	0.9	2.0	0.9	*	***	***
I am among the … lonely people (1–4)	1.2	0.5	1.6	0.8	1.9	0.7	***	***	***
Female (vs. male)	0.54		0.39		0.44		**		
Age (55–66)	60.6	3.1	60.9	2.9	60.8	3.1			
Married (vs. not married and no partner)	0.70		0.81		0.78		*		
Number of persons in household (0–8)	0.3	0.6	2.1	1.8	0.7	1.0	***	***	***
Number of children (0–13)	1.8	1.3	4.7	2.3	3.5	1.6	***	***	***
Contact frequency with children/children-in-law (1–5)	3.3	1.6	3.9	1.4	4.2	1.0	***	***	*
Contact frequency with grandchildren (1–5)	2.3	1.5	3.2	1.5	3.4	1.4	***	***	
Contact frequency with other kin (1–5)	3.4	0.9	2.9	1.1	2.9	1.0	***	***	
Contact frequency with friends/acquaintances (1–5)	3.7	0.8	4.0	1.2	4.1	0.9	**	***	
Contact frequency with neighbours (1–5)	3.8	1.0	4.1	1.1	4.1	1.0	**	**	
Educational level (1–9)	5.6	2.2	2.5	2.1	2.6	1.8	***	***	
Income level (1–24)	13.6	5.7	7.5	3.1	7.8	3.2	***	***	
Satisfaction with income (1–5)	4.1	1.3	2.7	1.6	2.1	1.3	***	***	***
Employed (vs. non-employed)	0.58		0.30		0.20		***	***	
Membership in organisations (vs. no membership)	0.73		0.77		0.95			***	***
Internet use (vs. no use)	0.91		0.40		0.32		***	***	
Church/mosque attendance (1–6)	1.8	1.4	4.7	1.9	4.3	1.9	***	***	*
Mastery (5–25)	18.8	3.3	16.7	5.6	13.8	4.6	***	***	***
General health (1–5)	3.7	0.9	2.6	1.1	2.5	1.0	***	***	
Number of chronic diseases (0–6)	1.0	0.9	1.3	1.1	2.0	1.4	*	***	***
Physical functioning (6–30)	28.9	2.6	27.1	3.9	24.8	5.5	***	***	***
Cognitive functioning (0–30)	28.5	1.6	27.2	2.5	26.0	2.8	***	***	***
Depressive symptoms (0–60)	7.7	7.3	15.9	11.3	18.2	11.0	***	***	

*p < 0.05; **p < 0.01; ***p < 0.001; *N* = 703

In addition to gender (0 = man, 1 = woman) and age, categories of independent variables covered five domains: social relationships, socio-economic position, social participation, mastery and health. With regard to social relationships, marital status (0 = not married and no partner, 1 = married), number of persons in the household other than the respondent and a spouse (0–8) and number of children (0–13) were assessed. Contact frequency was asked for five types of relationships (Schellingerhout 2004): children/children-in-law outside the household, grandchildren outside the household, other family, friends and acquaintances, and neighbours and other people in the neighbourhood. For the last two, Moroccans and Turks answered for Moroccan and Turkish relationships, respectively, and for Dutch and other relationships. Across the two questions, the highest frequency was chosen. Answer options vary from ‘less than monthly’ (1) to ‘every day’ (5).

Socio-economic position was measured with three variables. Level of education varies from ‘no completed’ (1) to ‘university’ education (9). Monthly net income was measured as euros per month in 24 categories. Income satisfaction ranges from ‘dissatisfied’ (1) to ‘satisfied’ (5).

Social participation was measured in three dichotomous variables. People were asked whether they worked, whether they were members of an organisation such as a trade union or a political party, and whether they used the Internet. A fourth, ordinal variable is church or mosque attendance, with values ranging from ‘not a member or never attend’ (1) to ‘weekly or more often’ (6).

Mastery is the feeling of being able to control important conditions affecting one’s life. We presented five statements (Pearlin and Schooler 1978). An example is: ‘I have little control over the things that happen to me’. Answer options vary from ‘strongly disagree’ (1) to ‘strongly agree’ (5). Cronbach’s alpha was 0.83 for Moroccans, 0.80 for Turks and 0.79 for Dutch. Scale values are 5–25.

Five health variables were studied. Answers to the question of general health range from ‘poor’ (1) to ‘excellent’ (5). Chronic diseases are diseases and complaints that last at least three months, or for which people are being treated or monitored by a doctor for a long time. We count the number of diseases (maximum six). Six questions referred to physical activities in daily life, such as walking. Answer options range from ‘cannot perform the activity’ (1) to ‘the activity is performed without help’ (5). Cronbach’s alpha was 0.77 for Moroccans, 0.87 for Turks and 0.82 for Dutch. Scale values are 6–30. Cognitive functioning was measured using the Mini-Mental State Examination (Folstein et al. 1975), with values between ‘very poor’ (0) and ‘good’ (30). Twenty items were presented about depressive symptoms (Radloff 1977). Cronbach’s alpha was 0.92 for Moroccans, 0.91 for Turks and 0.90 for Dutch. Scale values are 0–60.

### Procedure

For Hypothesis 1, we investigated psychometric scale characteristics within the three origin categories. Loevinger’s homogeneity indicates the correlation between the item scores (lower limit 0.30) (Mokken 1971). Reliability looks at the interrelationship in terms of number of items (lower limit 0.80) (Nunnally and Bernstein 1994). For reliability, we calculated Cronbach’s alpha and an improved version of it called the ‘greatest lower bound’ (GLB) (Bendermacher 2010). There is concurrent validity when a measurement is sufficiently related to other variables that are likely to measure the same concept (de Groot 1961). We calculated the correlation between the scale score and the answers to the two direct loneliness questions (Spearman’s rho; from 0.50 the correlation is seen as moderate to strong). Bias (‘differential item functioning’) was tested by verifying, for each item in the pooled sample, whether the degree of consent given in the origin categories corresponded with the scale score (uniform) and the increasing chance of consent for an increasing scale score (non-uniform) (Osterlind and Everson 2009). Logistic regression of the dichotomised item score was performed with origin and scale score as interacting predictors. The Wald statistic is Chi-square distributed and sensitive to sample size. Predicted values were used to determine item characteristic curves.

By means of variance analysis with the Bonferroni correction, we examined whether risk factors affect migrants more often compared to the Dutch. For testing Hypothesis 2, we applied ordinary linear regression analysis of loneliness in SPSS and investigated which factors are associated with loneliness. Tolerance testing indicates that all independent variables qualified for the regression analysis assumption concerning the absence of multicollinearity. We imputed missing values (none for loneliness; 1% for household size, contact with other kin, satisfaction with income level, church/mosque attendance; 2% for contact with grandchildren; 10% for income level; 18% for cognitive functioning) and created twenty data sets, presenting the pooled estimates. We controlled for gender and age, entering the differences between the origin categories as dummy variables. Three sets of analyses were run, first examining whether levels of risk factors reduce the differences in average loneliness by origin, then looking at the effect of individual variables and at the effects in a multivariate model that included all risk factors.

To test Hypothesis 3, we assessed whether the protective effect of factors differs across categories of origin. We conducted stratified regression analysis in Mplus (Muthén and Muthen 2017) and applied Bayesian multiple imputation with twenty iterations. For ease of interpretation of intercept and effects, all variables are centred within the three categories. The unconstrained model has no constraints on coefficient equality across Dutch, Moroccans and Turks. In the constrained model, all parameters are constrained to be equal. We released the constraints for all predictor variables one by one. From these 23 models, we selected models with better fit than the constrained model. Model improvement was reviewed by the Wald-distributed change in − 2 log likelihood and the decrease in Akaike information criterion (AIC), Bayesian information criterion (BIC) and sample-size adjusted BIC; any decrease points to model improvement. The final model only includes equality constraints for predictor variables whose release did not show model improvement. For the other predictor variables, we tested coefficient equality across Dutch, Moroccans and Turks by calculating the *z*-statistic (Clogg et al. 1995). For a further test of model fit, we used the comparative fit index (CFI), the root mean square error of approximation (RMSEA) and the standardised root mean squared residual (SRMR). CFI ≥ 0.95, RMSEA ≤ 0.06 and SRMR ≤ 0.08 support acceptable model fit (Hu and Bentler 1999).

## Results

### Loneliness scale validity

**Fig. 1:**
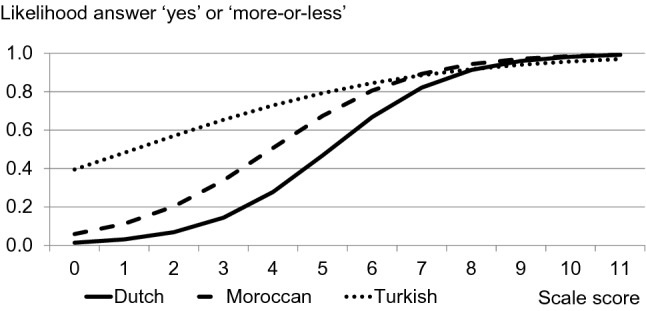
Item characteristic curve of ‘I miss the pleasure of the company of others’ by origin

The homogeneity and reliability of the item set within the origin categories are sufficient (Table 2). The correlation between the loneliness scale and the two direct questions indicates concurrent validity. For most characteristics, the values in the three origin categories were close to each other. The exception is the lower homogeneity among Moroccans. For five items, there was no bias: ‘I miss having a really close friend’, ‘I find my circle of friends and acquaintances too limited’, ‘There are many people I can trust completely’, ‘I miss having people around me’ and ‘I often feel rejected’. There was no significant Wald for either main effect of origin or interaction effect (not in the table). The biggest problem was found in the item ‘I miss the pleasure of the company of others’. The predicted chances of agreeing to this item are relatively high for Turks, and to a lesser extent for Moroccans (Fig. 1). For example, with a score of 3 points on the loneliness scale the probability of agreeing is 0.65 for Turks and 0.34 for Moroccans, compared to 0.14 for the Dutch (Table 3). This response tendency is also seen in ‘I experience a general sense of emptiness’. Turks and Moroccans scored relatively high in the direction of loneliness (so they often denied the item) on the item ‘There is always someone I can talk to about my day-to-day problems’. Moroccans scored relatively high in the direction of loneliness on the items ‘I can call on my friends whenever I need them’, ‘There are enough people I feel close to’ (together with the Dutch) and ‘There are plenty of people I can lean on when I have problems’. We find support for Hypothesis 1 that the loneliness scale is a valid instrument in this study, yet item-specific bias should also be considered.

**Table 2:** Homogeneity, reliability and congruent validity of the loneliness scale by origin

	Dutch	Moroccan	Turkish
Loevinger’s homogeneity (H)	0.48	0.30	0.44
Reliability (Cronbach’s alpha)	0.87	0.80	0.84
Reliability (GLB)	0.92	0.87	0.89
I sometimes feel lonely (Spearman’s rho)	0.53	0.61	0.61
I am among the … lonely people (Spearman’s rho)	0.51	0.58	0.55

*N* = 703

**Table 3:** Bias of loneliness item scores by origin

	Uniform		Non-uniform		Likelihood of agreeing when scale score = 3		
	Moroccan	Turkish	Moroccan	Turkish			
	Wald	Wald	Wald	Wald	Dutch	Moroccan	Turkish
There is always someone I can talk to about my day-to-day problems	6.8**	6.0*	3.3	7.0**	0.18	0.30	0.25
I miss having a really close friend	0.0	3.3	2.8	0.7	0.29	0.19	0.42
I experience a general sense of emptiness	2.7	7.7**	0.6	1.3	0.09	0.19	0.28
There are plenty of people I can lean on when I have problems	11.1***	0.2	7.3**	0.1	0.08	0.22	0.07
I miss the pleasure of the company of others	5.3*	47.1***	0.7	13.5***	0.14	0.34	0.65
I find my circle of friends and acquaintances too limited	2.3	1.3	4.6*	4.0*	0.29	0.27	0.25
There are many people I can trust completely	0.6	0.1	4.4*	4.5*	0.44	0.21	0.31
There are enough people I feel close to	2.5	4.1*	2.2	0.6	0.20	0.25	0.08
I miss having people around me	2.7	0.2	2.8	1.2	0.19	0.24	0.17
I often feel rejected	0.0	0.1	0.1	0.0	0.05	0.05	0.04
I can call on my friends whenever I need them	16.2***	3.4	8.0**	0.0	0.12	0.32	0.03

*p < 0.05; **p < 0.01; ***p < 0.001; *N* = 703

### Differences in risk and protective factors

There were major differences between the three origin categories in the five domains. Moroccans and Turks are more socially embedded than the Dutch, except for contact frequency with other kin (Table 1). Moroccans have more children, persons in the household and contact with children/children-in-law than Turks. Migrants have a relatively weak socio-economic position, with a lower education, lower income and, especially the Turks, less satisfaction with their income than the Dutch. In terms of social participation, migrants are less likely to work and be on the Internet. On the other hand, they attend mosque more often than the Dutch go to church, and more Turks are members of organisations. Migrants, especially Turks, experience less mastery and are in poorer health on all aspects compared to the Dutch.

### Explanation of differences in loneliness

On the basis of the differences in loneliness-related factors, the expectation is that the stronger loneliness among older Moroccan and Turkish migrants cannot be explained by differences in social relationships but by their weaker situation in the four other domains. The results of regression analysis support Hypothesis 2 (Table S1 of the Supplementary Material). Controlled for age and gender (both not significant), the loneliness of the Dutch is 1.7 points on a scale of 0–11. The loneliness of Moroccans is 2.8 points higher and that of Turks 3.9 points higher (Model 1). If we take into account the (better) position in the domain of social relationships, the loneliness of Moroccans and Turks is 3.0 and 4.3 points higher, respectively (Model 2). Taking into account differences in socio-economic position, the difference in loneliness is 1.5 and 2.4 points, respectively (Model 3). After adding only variables for social participation, the difference in loneliness is 2.4 and 3.4 points, respectively (Model 4). When we model the differences in mastery, the loneliness of Moroccans and Turks is 2.1 and 2.4 points higher, respectively (Model 5). Finally, controlled for health factors alone, the difference in loneliness is 1.2 and 2.0 points, respectively (Model 6). Each model adjustment is significant (p < 0.001). The weaker socio-economic position, lower social participation and mastery and poorer health thus contribute significantly to the stronger loneliness among Moroccans and Turks. If we include all factors simultaneously in the regression model (Table 4), on average Moroccans are 1.2 points and Turks 1.9 points lonelier than the Dutch. This multivariate model shows that more than half of the difference in loneliness originally found between the origin categories (2.8 for Moroccans and 3.9 points for Turks) can be attributed to differences in the risk factors studied.

**Table 4:** Regression of loneliness (range 0–11)

	Controlled for gender, age, origin		Multivariate	
	B	SE B	B	SE B
Constant			2.80	0.20***
Female (vs. male)			− 0.45	0.20*
Age (55–66)			0.04	0.03
Moroccan (vs. Dutch)			1.21	0.36***
Turkish (vs. Dutch)			1.87	0.32***
Married (vs. not married and no partner)	− 1.98	0.25***	− 1.09	0.26***
Number of persons in household (0–8)	− 0.10	0.10	0.04	0.09
Number of children (0–13)	− 0.12	0.07	0.03	0.07
Contact frequency with children/children-in-law (1–5)	− 0.34	0.08***	− 0.16	0.08
Contact frequency with grandchildren (1–5)	− 0.23	0.08**	− 0.10	0.08
Contact frequency with other kin (1–5)	− 0.35	0.11**	− 0.14	0.09
Contact frequency with friends/acquaintances (1–5)	− 0.58	0.11***	− 0.31	0.10**
Contact frequency with neighbours (1–5)	− 0.45	0.11***	− 0.16	0.09
Educational level (1–9)	− 0.11	0.06*	− 0.05	0.05
Income level (1–24)	− 0.18	0.03***	0.00	0.03
Satisfaction with income (1–5)	− 0.61	0.08***	− 0.29	0.07***
Employed (vs. non-employed)	− 1.16	0.25***	− 0.07	0.23
Membership in organisations (vs. no membership)	− 0.50	0.30	− 0.04	0.25
Internet use (vs. no use)	− 0.89	0.28**	0.24	0.25
Church/mosque attendance (1–6)	− 0.10	0.07	0.04	0.06
Mastery (5–25)	− 0.30	0.02***	− 0.13	0.02***
General health (1–5)	− 0.89	0.11***	− 0.08	0.11
Number of chronic diseases (0–6)	0.41	0.10***	− 0.12	0.09
Physical functioning (6–30)	− 0.18	0.03***	0.00	0.03
Cognitive functioning (0–30)	− 0.09	0.06	0.03	0.05
Depressive symptoms (0–60)	0.17	0.01***	0.11	0.01***

*p < 0.05; **p < 0.01; ***p < 0.001. *N* = 703. Multivariate model: *R*^2^ = 0.57

The parameters of the regression of loneliness are shown in Table 4. The left-hand section of the table shows the effects, controlled for gender and age, and after entering the differences between the three origin categories. All effects are in the expected direction, and most are statistically significant. The multivariate model in the right-hand section points to the significance of five factors. Being married reduces feelings of loneliness. Because in the sample migrants are married more often than the Dutch, the average loneliness among Moroccans and Turks is 0.1 points lower. Moroccans and Turks also have more contact with friends and acquaintances, which gives 0.1 points reduction in loneliness. Three factors do increase the average loneliness of migrants: they are more dissatisfied with their income (resulting in an increase in loneliness score of 0.4 points for Moroccans and 0.6 points for Turks), have less mastery (0.3 and 0.7 point increase, respectively) and have more depressive symptoms (0.9 and 1.2 point increase, respectively).

### Difference in protective effects of predictor variables

Model fit statistics for the prediction of loneliness stratified by origin are presented in Table S2. The unconstrained model has a perfect fit to the data by definition: CFI = 1.00, RMSEA = 0.00 and SRMR = 0.00. The model with all parameters constrained to be equal across the three categories has good fit to the data. Releasing the effects one by one from the constraint to be equal, we find model improvement for five predictors. The final model shows an improved fit over the constrained model: Wald = 2*-(1526.1–1546.4) = 40.5 (df = 10, *p *< 0.001), and AIC and sample-size adjusted BIC are lower. CFI = 1.00, RMSEA = 0.00 and SRMR = 0.01.

The effects of predictor variables on loneliness variation among Dutch, Moroccans and Turks are presented in Table 5. The results from the tests of coefficient equality (Hypothesis 3) show differences for four predictor variables only (the differences between the intercepts reflect differences in the means across origin categories). Marriage protects the Dutch more than Moroccans. Controlled for the other predictors, married Dutch are estimated to have a loneliness score 1.2 and non-married Dutch 2.8—a difference of 1.6 points. The difference is much smaller among Moroccans (0.4 points, with loneliness scores of 4.4 and 4.8, respectively), and among Turks (0.8 points, with loneliness scores of 5.5 and 6.3, respectively). A higher contact frequency with children and children-in-law protects Turks, but not Moroccans or Dutch. For Turks without children or less than monthly contact with their children/children-in-law, which applies to 7% of Turks only, the estimated loneliness score is 7.3 and for everyday contact (46% of Turks) it is 5.3. The estimates for Dutch and Moroccans hardly vary with contact frequency. A higher educational level protects Moroccans (0.8 points lower in loneliness score; not significant) and Turks (2.1 points lower); Dutch with a higher educational level score 0.9 more points in loneliness than Dutch with a lower educational level. Better physical functioning protects the Dutch (a 1.1-point advantage compared to those with poor physical functioning) better than Turks (a non-significant 0.3-point disadvantage); Moroccans with a high level of physical functioning have a non-significant advantage of 0.2 points.

**Table 5:** Regression of loneliness (range 0–11) stratified by origin

	Dutch			Moroccan			Turkish			Dutch versus Moroccan	Dutch versus Turkish	Moroccans versus Turkish
	B	SE B	*t*	B	SE B	*t*	B	SE B	*t*	*z*	*z*	*z*
Constant	1.70	0.11	16.2***	4.51	0.16	28.1***	5.65	0.17	32.9***	− 14.7***	− 19.6***	− 4.9***
Female (vs. male)	− 0.64	0.18	− 3.6***	− 0.64	0.18	− 3.6***	− 0.64	0.18	− 3.6***			
Age (55–66)	0.04	0.03	1.3	0.04	0.03	1.3	0.04	0.03	1.3			
Married (vs. not married and no partner)	− 1.59	0.31	− 5.2***	− 0.37	0.45	− 0.8	− 0.83	0.44	− 1.9	− 2.2*	− 1.4	0.7
Number of persons in household (0–8)	0.04	0.09	0.4	0.04	0.09	0.4	0.04	0.09	0.4			
Number of children (0–13)	− 0.04	0.07	− 0.6	− 0.04	0.07	− 0.6	− 0.04	0.07	− 0.6			
Contact frequency with children/children-in-law (1–5)	0.04	0.08	0.5	0.02	0.14	0.2	− 0.50	0.18	− 2.8**	0.1	2.8**	2.3*
Contact frequency with grandchildren (1–5)	− 0.08	0.07	− 1.1	− 0.08	0.07	− 1.1	− 0.08	0.07	− 1.1			
Contact frequency with other kin (1–5)	− 0.12	0.09	− 1.4	− 0.12	0.09	− 1.4	− 0.12	0.09	− 1.4			
Contact frequency with friends/acquaintances (1–5)	− 0.37	0.09	− 4.0***	− 0.37	0.09	− 4.0***	− 0.37	0.09	− 4.0***			
Contact frequency with neighbours (1–5)	− 0.14	0.09	− 1.6	− 0.14	0.09	− 1.6	− 0.14	0.09	− 1.6			
Educational level (1–9)	0.15	0.06	2.5*	− 0.13	0.09	− 1.4	− 0.35	0.11	− 3.2**	2.6**	4.0***	1.6
Income level (1–24)	0.00	0.03	0.1	0.00	0.03	0.1	0.00	0.03	0.1			
Satisfaction with income (1–5)	− 0.25	0.07	− 3.8***	− 0.25	0.07	− 3.8***	− 0.25	0.07	− 3.8***			
Employed (vs. non-employed)	− 0.15	0.20	− 0.7	− 0.15	0.20	− 0.7	− 0.15	0.20	− 0.7			
Membership in organisations (vs. no membership)	0.00	0.22	0.0	0.00	0.22	0.0	0.00	0.22	0.0			
Internet use (vs. no use)	0.36	0.25	1.4	0.36	0.25	1.4	0.36	0.25	1.4			
Church/mosque attendance (1–6)	0.02	0.05	0.3	0.02	0.05	0.3	0.02	0.05	0.3			
Mastery (5–25)	− 0.14	0.02	− 5.8***	− 0.14	0.02	− 5.8***	− 0.14	0.02	− 5.8***			
General health (1–5)	0.00	0.10	0.0	0.00	0.10	0.0	0.00	0.10	0.0			
Number of chronic diseases (0–6)	0.04	0.13	0.3	− 0.27	0.16	− 1.7	− 0.12	0.15	− 0.8	1.5	0.8	− 0.7
Physical functioning (6–30)	− 0.12	0.05	− 2.6**	− 0.02	0.05	− 0.4	0.04	0.04	0.9	− 1.5	− 2.5*	− 0.9
Cognitive functioning (0–30)	0.03	0.05	0.6	0.03	0.05	0.6	0.03	0.05	0.6			
Depressive symptoms (0–60)	0.12	0.01	10.1***	0.12	0.01	10.1***	0.12	0.01	10.1***			

*p < 0.05; **p < 0.01; ***p < 0.001; *N* = 703

## Discussion

Older migrants of Moroccan origin and in particular of Turkish origin are on average lonelier than their Dutch age peers. This has already been observed in the four big Dutch cities (el Fakiri and Bouwman-Notenboom 2015; Uysal-Bozkir et al. 2017), and the results of this research in fifteen cities confirm it. The now-established psychometric characteristics of the loneliness scale of De Jong Gierveld denote that the scale within each origin category is very useful. The test of concurrent validity indicates that the loneliness scale has a good resemblance with direct measurements of loneliness. For comparison between the categories, it is important that the item characteristic curves in the three origin categories be approximately the same. For six items, the similarity was statistically insufficient. This large number is inseparable from sample size and should therefore not be taken as starting point for an assessment. In qualitative research (Torensma 2014), one of these six items and another item were described as interculturally sensitive. The item about pleasure of social company (‘conviviality’), where we found the biggest problem, was not found to be problematic in that study. Hence, there are clear signs that some items have different weights for the three origin categories, and further research is desirable. But because the scale consists of different items, item-specific biases may compensate each other in the sum score. Research into homogeneity, reliability, concurrent validity and bias currently provides sufficient indications of the validity of the scale as a whole and does not preclude research comparing intensity of loneliness between different categories of origin.

In the field of social relationships, older migrants are better protected than older Dutch adults, at least in terms of availability of and contact within relationships. No information was available on other aspects such as content of the relationships, which might explain why marriage appears to protect the Dutch more than Moroccans and why frequent contact with children and children-in-law is most protective for Turks. Previous research (Visser and el Fakiri 2016) did examine the significance for loneliness of a partner relationship and contact with neighbours, but not the significance of other types of social relationships. Another Dutch study (ten Kate et al. 2020) among middle-aged and older non-Western migrants from five different countries together showed that their lower relationship satisfaction compared to Dutch people was related to higher loneliness. Relationship satisfaction, however, is conceptually close to loneliness, and relationship content was not measured.

In line with the expectation, we found that Dutch older migrants are at a greater risk in the domains of socio-economic position, social participation, health and mastery, and that this is related to their average stronger loneliness. Moreover, older migrants experience more severe consequences from a lower educational level (Moroccans and Turks) and poorer physical functioning (Turks) than non-migrants. For the first three domains, this corresponds with the results of earlier Dutch research among migrants (Visser and el Fakiri 2016). These four domains also appeared to be important in research into ageing among older Dutch adults, but only from the age of 70 (van Tilburg et al. 2018). The comparison of older Moroccans and Turks aged around 60 with their Dutch age peers, ‘young-olds’, is less appropriate.

We only examined risk factors for which data were available in both data sets. The LASA study among older Dutch adults was not designed for comparison with migrants. For example, poor Dutch language proficiency may pose a risk of loneliness for migrants (Klok et al. 2017), but it is unknown whether language proficiency is a factor in the development or persistence of loneliness among respondents of Dutch origin. Migrant-specific factors cannot be included in the comparison with the Dutch. For example, transnational behaviour increases the risk of loneliness (Klok et al. 2017). Because migrants want to keep their own identity and culture, they are focused on their home country next to their life in the Netherlands. For a long time, their social life in the Netherlands was not a priority because there was a prospect of return. Factors such as social exclusion, discrimination and insufficient accessibility to regular professional care have not been investigated. Neither could we pay any attention to differences between the categories of origin with respect to their views on the priority they give to their individual goals versus those of the collective they are part of. Dutch culture was already highly individualistic decades ago compared to Morocco and Turkey (Hofstede 1983), and this may mean that older Dutch adults have different expectations and are less likely to feel lonely than older Moroccans and Turks (Swader 2019). The present study therefore gives a very limited view on cross-cultural factors in loneliness and has a limited view on the diversity within the three origin categories.

The increased vulnerability of migrants ensues from a combination of three circumstances: they have experienced the stressful event of having left their home country and are growing old in a second homeland where they are a minority (Dowd and Bengtson 1978). Migration caused a cultural shock (Oberg 1960), as they were confronted with behaviours, customs, beliefs and a food culture, landscape, climate and language that were different. Adaptation and alignment are stressful (Berry et al. 1987), and stress increases the risk of loneliness. Migrants are a disadvantaged minority that is treated as such. In the Netherlands, there are many negative prejudices against Moroccan and Turkish migrants (Coenders et al. 2015; Pettigrew et al. 1997). In addition to the factors investigated that provide important explanations for the differences in loneliness between migrants and older Dutch adults, personal migration history and experiences of deprivation and ethnic discrimination could also plausibly contribute to their loneliness.

We suggest that practitioners, in particular those who develop and execute loneliness interventions, may profit from the results. Many loneliness interventions focus on increasing the possibilities for meeting other people, personal contact and practical support (Gardiner et al. 2018). This choice can be followed from the definition of loneliness where the lack of personal relationships is central. From the results of this research, it is clear that this type of intervention does not sufficiently address the loneliness problem of many older migrants. Several factors have proven to be important in other domains, namely weak socio-economic position, low level of social participation, lack of mastery and copious depressive symptoms. This is indicative of the need to explore additional solutions. Examples include focusing on meaningful activities, strengthening the experience of having a socially valued role, and avoiding negative interpretations.

We found that many but not all protective effects of loneliness-associated characteristics are universal across the three categories. For example, many Turks have frequent contact with children and children-in-law, but for those who have none, improving contact frequency may be beneficial. The similarity in risk factors among older Dutch and older migrants suggests that an approach which is potentially effective among older adults of Dutch origin can also be applied to older migrants. Such an intervention approach may need to be adapted to migrants in order to be successful. It is also necessary to determine which migrant-specific factors need to be taken into account in the approach.

## Electronic supplementary material

Below is the link to the electronic supplementary material.

Supplementary material 1 (DOCX 37 kb)
