# Supplementary material for: Stronger feelings of loneliness among Moroccan and Turkish older adults in the Netherlands: in search for an explanation
Source: Eur J Ageing. 2020 Feb 22;18(3):311–22. doi: 10.1007/s10433-020-00562-x (PMC8377113; doi:10.1007/s10433-020-00562-x)
Supplement: Supplementary file 1 — Supplementary material 1 (DOCX 37 kb) [file 10433_2020_562_MOESM1_ESM.docx]

Stronger feelings of loneliness among Moroccan and Turkish older adults in the Netherlands: In search for an explanation

Supplementary Material

Table S1. Regression of loneliness (range 0-11)

|  | Model 1 | | | Model 2 | | | Model 3 | | | Model 4 | | | Model 5 | | | Model 6 | | |
| --- | --- | --- | --- | --- | --- | --- | --- | --- | --- | --- | --- | --- | --- | --- | --- | --- | --- | --- |
|  | B | SE B |  | B | SE B |  | B | SE B |  | B | SE B |  | B | SE B |  | B | SE B |  |
| Constant | 1.70 | 0.17 | *** | 1.54 | 0.19 | *** | 2.56 | 0.21 | *** | 1.99 | 0.22 | *** | 2.38 | 0.16 | *** | 2.74 | 0.17 | *** |
| Female (vs. male) | 0.02 | 0.22 |  | -0.34 | 0.23 |  | -0.06 | 0.22 |  | -0.40 | 0.24 |  | -0.22 | 0.20 |  | -0.48 | 0.19 | * |
| Age (55-66) | 0.01 | 0.04 |  | 0.02 | 0.04 |  | -0.02 | 0.04 |  | -0.04 | 0.04 |  | 0.01 | 0.03 |  | 0.04 | 0.03 |  |
| Moroccan (vs. Dutch) | 2.82 | 0.28 | *** | 3.02 | 0.35 | *** | 1.49 | 0.34 | *** | 2.39 | 0.36 | *** | 2.15 | 0.26 | *** | 1.22 | 0.27 | *** |
| Turkish (vs. Dutch) | 3.95 | 0.26 | *** | 4.29 | 0.28 | *** | 2.37 | 0.33 | *** | 3.40 | 0.35 | *** | 2.42 | 0.26 | *** | 2.03 | 0.27 | *** |
| Married (vs. not married and no partner) |  |  |  | -1.86 | 0.26 | *** |  |  |  |  |  |  |  |  |  |  |  |  |
| Number of persons in household (0-8) |  |  |  | 0.00 | 0.10 |  |  |  |  |  |  |  |  |  |  |  |  |  |
| Number of children (0-13) |  |  |  | 0.05 | 0.08 |  |  |  |  |  |  |  |  |  |  |  |  |  |
| Contact frequency with children/children-in-law (1-5) |  |  |  | -0.27 | 0.10 | ** |  |  |  |  |  |  |  |  |  |  |  |  |
| Contact frequency with grandchildren (1-5) |  |  |  | 0.02 | 0.09 |  |  |  |  |  |  |  |  |  |  |  |  |  |
| Contact frequency with other kin (1-5) |  |  |  | -0.24 | 0.11 | * |  |  |  |  |  |  |  |  |  |  |  |  |
| Contact frequency with friends/acquaintances (1-5) |  |  |  | -0.46 | 0.12 | *** |  |  |  |  |  |  |  |  |  |  |  |  |
| Contact frequency with neighbours (1-5) |  |  |  | -0.19 | 0.11 |  |  |  |  |  |  |  |  |  |  |  |  |  |
| Educational level (1-9) |  |  |  |  |  |  | 0.00 | 0.06 |  |  |  |  |  |  |  |  |  |  |
| Income level (1-24) |  |  |  |  |  |  | -0.12 | 0.03 | *** |  |  |  |  |  |  |  |  |  |
| Satisfaction with income (1-5) |  |  |  |  |  |  | -0.46 | 0.09 | *** |  |  |  |  |  |  |  |  |  |
| Employed (vs. non-employed) |  |  |  |  |  |  |  |  |  | -1.07 | 0.26 | *** |  |  |  |  |  |  |
| Membership in organisations (vs. no membership) |  |  |  |  |  |  |  |  |  | -0.36 | 0.30 |  |  |  |  |  |  |  |
| Internet use (vs. no use) |  |  |  |  |  |  |  |  |  | -0.78 | 0.28 | ** |  |  |  |  |  |  |
| Church/mosque attendance (1-6) |  |  |  |  |  |  |  |  |  | -0.10 | 0.07 |  |  |  |  |  |  |  |
| Mastery (5-25) |  |  |  |  |  |  |  |  |  |  |  |  | -0.30 | 0.02 | *** |  |  |  |
| General health (1-5) |  |  |  |  |  |  |  |  |  |  |  |  |  |  |  | -0.19 | 0.12 |  |
| Number of chronic diseases (0-6) |  |  |  |  |  |  |  |  |  |  |  |  |  |  |  | -0.07 | 0.09 |  |
| Physical functioning (6-30) |  |  |  |  |  |  |  |  |  |  |  |  |  |  |  | -0.02 | 0.03 |  |
| Cognitive functioning (0-30) |  |  |  |  |  |  |  |  |  |  |  |  |  |  |  | 0.02 | 0.05 |  |
| Depressive symptoms (0-60) |  |  |  |  |  |  |  |  |  |  |  |  |  |  |  | 0.16 | 0.01 | *** |
| R^2^ | 0.26 |  |  | 0.37 |  |  | 0.34 |  |  | 0.30 |  |  | 0.41 |  |  | 0.49 |  |  |
| * p<0.05; ** p<0.01; *** p<0.001. N=703. | | | | | | | | | | | | | | | | | | |

Table S2. Model fit of stratified regression of loneliness

|  | df | LL | AIC | BIC | Adj. BIC | CFI | RMSEA | SRMR | |
| --- | --- | --- | --- | --- | --- | --- | --- | --- | --- |
| Unconstrained | 75 | -1512.4 | 3174.8 | 3516.5 | 3278.3 | 1.00 | 0.00 | 0.00 | |
| Constrained | 29 | -1546.4 | 3150.8 | 3282.9 | 3190.8 | 0.97 | 0.03 | 0.01 | |
| Equality of effect released |  |  |  |  |  |  |  |  | |
| Female | 31 | -1546.2 | 3154.5 | 3295.7 | 3197.3 | 0.96 | 0.04 | 0.01 | |
| Age | 31 | -1546.2 | 3154.4 | 3295.6 | 3197.2 | 0.96 | 0.04 | 0.01 | |
| Married | 31 | -1542.2 | 3146.4 | 3287.6 | 3189.2 | 0.98 | 0.03 | 0.01 | |
| Number of persons in household | 31 | -1546.3 | 3154.7 | 3295.9 | 3197.5 | 0.96 | 0.04 | 0.01 | |
| Number of children | 31 | -1545.8 | 3153.6 | 3294.8 | 3196.4 | 0.96 | 0.04 | 0.01 | |
| Contact frequency with children/children-in-law | 31 | -1544.1 | 3150.1 | 3291.3 | 3192.9 | 0.97 | 0.03 | 0.01 | |
| Contact frequency with grandchildren | 31 | -1546.3 | 3154.6 | 3295.8 | 3197.4 | 0.96 | 0.04 | 0.01 | |
| Contact frequency with other kin | 31 | -1546.0 | 3154.0 | 3295.2 | 3196.8 | 0.96 | 0.04 | 0.01 | |
| Contact frequency with friends/acquaintances | 31 | -1545.4 | 3152.8 | 3294.0 | 3195.6 | 0.97 | 0.04 | 0.01 | |
| Contact frequency with neighbours | 31 | -1544.4 | 3150.8 | 3292.0 | 3193.6 | 0.97 | 0.03 | 0.01 | |
| Educational level | 31 | -1540.5 | 3143.1 | 3284.3 | 3185.9 | 0.99 | 0.02 | 0.01 | |
| Income level | 31 | -1544.9 | 3151.8 | 3293.0 | 3194.5 | 0.97 | 0.03 | 0.01 | |
| Satisfaction with income | 31 | -1546.3 | 3154.6 | 3295.8 | 3197.4 | 0.96 | 0.04 | 0.01 | |
| Employed | 31 | -1545.2 | 3152.3 | 3293.5 | 3195.1 | 0.97 | 0.03 | 0.01 | |
| Membership in organisations | 31 | -1546.0 | 3153.9 | 3295.1 | 3196.7 | 0.96 | 0.04 | 0.01 | |
| Internet use | 31 | -1546.2 | 3154.5 | 3295.7 | 3197.3 | 0.96 | 0.04 | 0.01 | |
| Church/mosque attendance | 31 | -1545.2 | 3152.4 | 3293.6 | 3195.2 | 0.97 | 0.04 | 0.01 | |
| Mastery | 31 | -1546.1 | 3154.2 | 3295.5 | 3197.0 | 0.96 | 0.04 | 0.01 | |
| General health | 31 | -1544.8 | 3151.6 | 3292.8 | 3194.3 | 0.97 | 0.03 | 0.01 | |
| Number of chronic diseases | 31 | -1543.8 | 3149.6 | 3290.8 | 3192.3 | 0.97 | 0.03 | 0.01 | |
| Physical functioning | 31 | -1542.8 | 3147.7 | 3288.9 | 3190.4 | 0.98 | 0.03 | 0.01 | |
| Cognitive functioning | 31 | -1545.5 | 3153.1 | 3294.3 | 3195.9 | 0.96 | 0.04 | 0.01 | |
| Depressive symptoms | 31 | -1545.3 | 3152.7 | 3293.9 | 3195.5 | 0.97 | 0.04 | 0.01 | |
| Final Model | 39 | -1526.1 | 3130.2 | 3307.9 | 3184.1 | 1.00 | 0.00 | 0.01 | |
| Note. df=degrees of freedom; LL=Log likelihood; AIC=Akaike Information Criterion; BIC=Bayesian Information Criterion; Adj. BIC= Sample-Size Adjusted BIC; RMSEA=Root Mean Square Error of Approximation; CFI=Comparative Fit Index; SRMR=Standardized Root Mean Square Residual | | | | | | | | |  |
